# Comprehensive review of diabetic ketoacidosis: an update

**DOI:** 10.1097/MS9.0000000000000894

**Published:** 2023-05-23

**Authors:** Chukwuka Elendu, Johnson A. David, Abasi-O. Udoyen, Emmanuel O. Egbunu, Ifeanyichukwu C. Ogbuiyi-Chima, Lilian O. Unakalamba, Awotoye I. Temitope, Jennifer O. Ibhiedu, Amos O. Ibhiedu, Promise U. Nwosu, Mercy O. Koroyin, Chimuanya Eze, Adeyemo I. Boluwatife, Omotayo Alabi, Olisa S. Okabekwa, John O. Fatoye, Habiba I. Ramon-Yusuf

**Affiliations:** a Federal Medical Center, Owerri; b University of Nigeria Teaching Hospital, Ituku Ozalla; c Redeemer’s University; d University of Ilorin Teaching Hospital; e Babcock University; f Rivers State University Teaching Hospital, Portharcourt; g Lagos University Teaching Hospital; h Abia State University, Nigeria; i VN Karazin National University, Kharkiv, Ukraine; j Pirogov Medical University, Rossijskij nacional’nyj issledovatel’skij medicinskij universitet imeni N I Pirogova, Russia; k Bromley Healthcare; l University Hospital Lewisham, UK

**Keywords:** diabetic ketoacidosis, hyperglycemia, insulin deficiency, ketones, type 1 diabetes

## Abstract

The most frequent hyperglycemic emergency and the leading cause of death in people with diabetes mellitus is diabetic ketoacidosis (DKA). DKA is common in people with type 1 diabetes, while type 2 diabetes accounts for roughly one-third of occurrences. Although DKA mortality rates have generally decreased to low levels, they are still significant in many underdeveloped nations. In industrialized countries, its mortality rate ranges from 2 to 5%, but in underdeveloped nations, it ranges from 6 to 24%. Therefore, it is always lethal if misdiagnosed or improperly treated. According to specific research, DKA can be present at the time of type 1 diabetes onset in 25 to 30% of cases and in 4 to 29% of young people with type 2 diabetes mellitus, and its features include hyperglycemia, metabolic acidosis, and ketosis with its triggering factors commonly being infections, newly discovered diabetes, and failure to start insulin therapy. Less than 20% of DKA patients present comatose, and patients with different levels of consciousness can present at other times. A close association between abnormalities found during a mental status evaluation and osmolality seems to exist. Hospital admission is necessary for vigorous intravenous fluid therapy, insulin therapy, electrolyte replacement, diagnosis and treatment of the underlying triggers, and routine monitoring of the patient’s clinical and laboratory conditions to manage DKA properly. Appropriate discharge plans should include actions to prevent a DKA recurrence and the proper selection and administration of insulin regimens.

## Introduction and background

Highlights

- Diabetic ketoacidosis is the most frequent hyperglycemic emergency.
- Diabetic ketoacidosis is the leading cause of death in people with diabetes mellitus.
- DKA is common in people with type 1 diabetes.

Diabetic ketoacidosis is a grievous complication of diabetes that occurs when there is a lack of insulin in the body, resulting in elevated blood glucose levels and the production of ketones. Diabetic ketoacidosis is a medical emergency that requires immediate treatment, as it can lead to life-threatening complications such as cerebral edema, acute respiratory distress syndrome, and sepsis^1^. It is becoming more prevalent worldwide, particularly among type 1 diabetic children and adolescents^1^. While insulin therapy and patient education have improved, diabetic ketoacidosis remains a common cause of hospitalization and is associated with notable morbidity and mortality^2,3^. Among the immediate treatment options for severe diabetic ketoacidosis is a multidisciplinary approach that emphasizes correcting fluid and electrolyte imbalances, restoring insulin sensitivity, and preventing complications. Primary care aims to prevent the progression of diabetic ketoacidosis and minimize the risk of cerebral edema, a rare but potentially fatal complication^4,5^. A recent publication has shed light on the pathophysiology of diabetic ketoacidosis, including the role of insulin deficiency, altered glucose metabolism, and acid-base disturbances^3^. Technological advances and monitoring techniques have also allowed for more precise management of fluid and electrolyte imbalances, insulin therapy, and other adjunctive therapies^5^. The management of severe diabetic ketoacidosis is not without challenges. For example, insulin therapy can rapidly drop blood glucose levels, increasing the risk of hypoglycemia.

Similarly, fluid therapy can lead to fluid overload and other complications, particularly in patients with underlying comorbidities^2^. To adequately address these challenges, there is a need for a comprehensive review of immediate care for severe diabetic ketoacidosis^4^. Such a review would synthesize current research findings, provide evidence-based recommendations, and highlight areas where further research is needed.

### Objectives

This review aims to comprehensively analyze the current state of immediate care for severe diabetic ketoacidosis. It will cover the pathophysiology, clinical manifestations, and management of diabetic ketoacidosis, focusing on evidence-based interventions. Furthermore, the study will highlight the challenges associated with immediate care for severe diabetic ketoacidosis and provide recommendations for addressing these challenges. It will be an essential resource for healthcare professionals managing severe diabetic ketoacidosis. This review can improve patient outcomes and reduce the morbidity and mortality associated with severe diabetic ketoacidosis by providing evidence-based guidance.

## Review

### Methodology

This comprehensive review compiles and evaluates recurrent and dominant topics discussed in the literature regarding the epidemiology, pathophysiology, and emergent therapy for patients with diabetic ketoacidosis, a complication of type 1 diabetes (T1DM) and less frequently type 2 diabetes (T2DM). To attain the purpose of the study, the authors undertook an exhaustive and advanced Pubmed search. Pubmed is an electronic database that serves as a search engine and gives access to more than 35 million MEDLINE articles that can be cited (medical, biomedical, nursing, life sciences, etc.). We used Boolean operators to combine the search terms and phrases; epidemiology, pathophysiology, diagnosis, and emergent therapy for patients with diabetic ketoacidosis. We also entered the vital alternative terms into the search for a broader reach. The review was limited to articles published within the past decade (2012–2022) and was not restricted to a specific region. We used only English language articles from academic journals with peer review. In addition, the study included articles with both abstracts and full texts to facilitate screening. Thirty-seven articles popped up after the filters were applied. After reading through the abstracts of the initial results to screen for eligibility, six papers were eventually selected since they also provided the most recent discussions regarding the topic.

### Definition and epidemiology

Diabetic ketoacidosis is a severe acute metabolic complication of diabetes mellitus characterized by hyperglycemia, hyperketonaemia, and metabolic acidosis. Patients have high insulin requirements, eventually depleting their body insulin^5^. This insulin deficiency leads to an excessive breakdown of fat, resulting in an excessive build-up of ketone bodies. Despite improvements in diabetic patients’ self-care, DKA still accounts for 14% of all hospital admissions of diabetic patients and 16% of all deaths linked to diabetes^4^. DKA commonly exists in people with type 1 diabetes, and about 3% of type 1 diabetes patients initially present with DKA; the incidence is two episodes per 100 patient-years of diabetes. Patients with type 2 diabetes can also develop it, though this is less typical^6,7^. Although the incidence of diabetic ketoacidosis in developing nations is unknown, it may be greater than in developed countries^8^. Whites have a greater prevalence of type 1 diabetes, contributing to the higher incidence of DKA in this racial group. For unknown causes, females are slightly more likely than males to develop DKA. Young ladies with type 1 diabetes frequently experience recurrent DKA, which is primarily brought on by failing to administer insulin therapy^6,9^. DKA is much more frequent in young children and teenagers than in adults among people with type 1 diabetes; however, patients with diabetes may experience DKA at any age. Intervention is feasible between the onset of symptoms and the development of DKA, although numerous factors (such as ethnic minority, lack of health insurance, lower BMI, preceding infection, delayed treatment, etc.) influence the risk of DKA among children and young people^9^.

### Pathophysiology

The abnormal physiology seen in a diabetic patient with ketoacidosis is due to absolute or relative insulin deficiency with the rise in hormones that put the body in a catabolic state and cause insulin resistance leading to hyperglycemia, hyperketonemia, hyperosmolarity, and electrolyte imbalances. These hormones include; glucagon, growth hormone, and catecholamines (epinephrine and norepinephrine)^10^. The event that most commonly precipitates diabetic ketoacidosis is usually a loss of insulin activity or increased demand for insulin, which can occur due to missed insulin doses, improper administration of insulin, or the presence of infections in a diabetic patient^11^. It can lead to an inability to transport glucose intracellularly; when this occurs, most cells cannot utilize glucose for energy, so intracellular hunger and starvation begin. Most cells shift to free fatty acids (FFA) as an energy source^12,13^. Without insulin, there becomes a plethora of FFA in the bloodstream because insulin impedes the lipolysis of adipocytes into glycerol and FFA^8^. These abundantly circulating FFA are taken to the liver and transported to its mitochondria for oxidation; then, ketone bodies are formed, including beta-hydroxybutyrate, acetone, and acetoacetate. Insulin checks the biochemical process, but excessive ketone production results from insufficient insulin^14^. In uncomplicated diabetes or starvation, triglycerides usually predominate ketones. The ketones produced do not overwhelm the body’s ability to get rid of them, putting it in a state of ketosis^15^. Glucagon, catecholamines, cortisol, and growth hormone also significantly increase blood glucose through gluconeogenesis and glycogenolysis^15^. The release of these hormones can also be a response to stress, which can take the form of infections (i.e., urinary tract infections and respiratory tract infections, especially the lower tract); trauma; myocardial infarction; acute pancreatitis; burns; surgery; strokes; substance abuse; and so on^16^. These stressors cause a release in inflammatory cytokines that increase insulin counter-regulatory hormones like glucagon, catecholamines, cortisol, and growth hormone^17^. These hormones put the body in a catabolic state, causing more lipolysis and proteolysis to synthesize glucose, which worsens hyperglycemia^18^.

### Causes

The commonest precipitants of diabetic ketoacidosis are poor compliance with insulin therapy, infections, and a new diagnosis of diabetes^19^. The most common precipitating factor of diabetic ketoacidosis in type 1 diabetes patients is nonadherence to treatment, while infections are the most common precipitant in type 2 diabetes patients^20^. Other causes are vascular events (e.g., acute coronary syndrome, cerebrovascular accidents, critical limb ischemia, bowel ischemia, and shock); excessive alcohol intake; illicit drugs such as cocaine and methamphetamine; antipsychotic drugs, for example, clozapine, risperidone, and olanzapine^19^.

### Laboratory abnormalities and diagnosis

In 2003, the American Diabetes Association modified the diagnostic criteria of DKA by introducing severity categories of mild, moderate, and severe, as displayed in Table 1 ^2,21^.

**Table 1:** Diagnostic criteria and severity of diabetic ketoacidosis

	Mild	Moderate	Severe
Plasma glucose (mmol/l)	>13.9	>13.9	>13.9
Arterial pH	7.25–7.30	7.00–7.24	<7.00
Serum bicarbonate (mmol/l)	15–18	10–14.9	<10
Urine ketones	++	++	+++
Serum ketones	+++	+++	+++
Anion gap	>10	>12	>12
Sensorium	Alert	Alert/drowsy	Stupor/coma
Serum sodium	Normal	Low	Low
Serum potassium	Normal	High	High
Serum phosphate	Normal	High	High

Abnormal changes in laboratory values give a significant clue of what exactly happens in patients with suspected diabetic ketoacidosis^2,21–23^.

The diagnosis of DKA consists of a triad of hyperglycemia, ketonemia, and metabolic acidosis.

All patients with positive ketones, constitutional symptoms, or suspicion of DKA and significantly elevated blood glucose levels [>13.9 mmol/l (>250 mg/dl)] should have electrolytes and blood gases checked to look for an anion gap metabolic acidosis^2^. Significantly in type 1 diabetes, DKA can develop within hours if you stop insulin injections or an insulin pump malfunctions^21^. The new American Diabetes Association definition of DKA includes a blood glucose level of 13.9 mmol/l (250 mg/dl)^2^. Many studies show that DKA is infrequent at lower levels except in situations with poor oral intake or pregnancy^22^. It is also essential to consider DKA in differential diagnoses for patients with anion gap metabolic acidosis. Check serum glucose even when the patient has no history of diabetes^15^. Consider DKA if the serum glucose is more significant than 13.9 mmol/l (250 mg/dl), but an elevated glucose level alone is insufficient to diagnose DKA. Suspect DKA in patients with diabetes with a concurrent infection, stroke, myocardial infarction, or other serious illness^13^. These intercurrent illnesses should be sought and treated aggressively^13^. Similarly, it is vital to consider DKA when patients with diabetes experience nausea and vomiting, even if the blood sugar level is less than 13.9 mmol/l (250 mg/dl)^10^. Euglycemic DKA occurs more often in patients who have not eaten but continue taking insulin^12^. A blood sugar level of less than 13.9 mmol/l (250 mg/dl) occurs in 1–7% of reported DKA cases and seems more common in patients with hepatic dysfunction or in those who are in patients^11^. Several drugs, such as glucocorticoids or thiazides, are well-known causes of hyperglycemia that may lead to DKA. Clinicians should also consider DKA in patients taking atypical antipsychotic drugs who present with hyperglycemia^23^. Atypical antipsychotic drugs have increased the frequency of diabetes, glucose intolerance, and DKA. The healthcare provider should measure such patients’ anion gap and ketone levels. Another type of antipsychotic drug must be chosen to help resolve this complication^24,25^. The presentation of a patient with DKA varies substantially depending on the severity of the episode^5^. Mild or moderately ill patients may describe vague symptoms of fatigue, lethargy, poor appetite, or headache. In type 1 diabetes, the history of polyuria and polydipsia may be relatively recent, but in type 2 diabetes, these symptoms may have been building for weeks to months^8^. Nausea, vomiting, and abdominal pain are commonly seen in DKA and may be related to the combined effects of dehydration, hypokalemia, ketonemia, and delayed gastric emptying^24^. Signs of dehydration, including poor skin turgor, decreased axillary sweat, or postural hypotension, may be present on physical examination^2^. Kussmaul respirations (a pattern of deep breathing and hyperventilation in response to metabolic acidosis) may be present^5^. Patients’ breath may smell fruity due to increased acetone from ketonemia, but the absence of this finding does not rule out DKA. One examination aspect that can be confusing is abdominal tenderness, which may resolve with the treatment of DKA or reflect a more acute abdominal process that precipitates DKA^19^. Abdominal pain correlates with the level of acidosis^15^. The physical examination should identify potential precipitating factors, such as infections or cardiovascular events. Patients may have mental status changes ranging from mild lethargy to delirium or coma. The most severe cases have features like hypotension, tachycardia, and coma^20^. Capillary blood ketone measurement is a relatively new quantitative and enzymatic test that determines levels of 3-β-hydroxybutyrate, one of the three ketone bodies^13^. The equipment is similar to that patients use for home blood glucose determination, but it requires specific strips. However, checking capillary blood ketones is much more expensive than checking urine ketones, and further clinical studies are needed to define the most appropriate role for β-hydroxybutyrate monitoring^24^. If clinical suspicion of DKA is high, a negative urine dipstick for ketones does not exclude DKA. Clinicians should know that urine test sticks do not measure β-hydroxybutyrate, the predominant ketone. Acetoacetate measured on the dipstick may not be high until later during the illness^23^. Arterial blood gas assessment is generally considered the most reliable method to evaluate the degree of acidosis in DKA, but a venous pH may be a more practical alternative. The average anion gap is 7–9 mmol/l but is ~25 mmol/l in DKA^2^. Rarely do patients with DKA have mixed acidosis and alkalosis with a pH close to normal. However, this unexpected laboratory result should not affect the treatment of DKA^17^. Determination of the arterial blood gas may be optional. Most DKA guidelines indicate that hyperglycemia of more than 13.9 mmol/l is necessary for diagnosing DKA; however, this is not an absolute requirement, as there are reports about DKA without hyperglycemia^20^. DKA without hyperglycemia is reported chiefly during pregnancy and in patients with prolonged vomiting or starvation^9^. It can also occur in patients with liver failure or alcohol abusers^9^. Ketone bodies are produced in the liver from acetyl-CoA liberated during lipolysis from fatty acids. For DKA to develop, an absolute or relative insulin deficiency must be present. Three ketone bodies are produced: acetone (resulting in the fruity odor of DKA patients), acetoacetate, and β-hydroxybutyrate (β-OHB). β-OHB is the most prominent contributor to metabolic acidosis in patients with DKA^18^. Acetone does not contribute to acidosis and is not usually measured as such. Acetoacetate can be measured in the urine with a urine dipstick utilizing the nitroprusside reaction^10^. As DKA resolves, β-OHB is oxidized to acetoacetate. Therefore, if only a urine ketone dipstick procedure is done, it might give the impression that the condition is not improving. Blood ketones can be measured with a point of care (bedside) meter utilizing capillary finger prick blood^10^. This measures β-OHB directly and accurately^11,12^. The American Diabetic Association recommends that the blood ketone measurement of β-OHB is preferable to urine measurement for diagnosing and monitoring DKA^8^. An arterial pH of less than 7.3 should be present in diagnosing DKA. The measurement of pH or serum bicarbonate is essential for the diagnosis and estimation of the severity of DKA^4^. The pH is also an important measure to assess improvement and treatment adjustment. A venous pH determination would probably be sufficient unless respiratory function must also be evaluated^12^. The venous pH is, on average, 0.03 lower than the arterial pH^23^.

### Differential diagnosis

Diabetic ketoacidosis may have a diverse and complex presentation, which makes it share many similarities in the way and manner it presents compared to other common pathologies; hence, other common pathologies may mimic diabetic ketoacidosis^1^. It is, therefore, essential to rule out other pathologies with similar presentations whenever a case of diabetic ketoacidosis is suspected. Differentials include; starvation ketoacidosis, pancreatitis, alcoholic ketoacidosis, lactic acidosis, uremia, overdose on diabetic medication, hyperosmolar hyperglycemic nonketotic syndrome, and myocardial infarction^2^.

### Treatment

Therapy goals in patients with hyperglycemic crises include improving the circulatory volume and tissue perfusion, gradual reduction of serum glucose and osmolality, correcting electrolyte imbalance, and identifying and promptly treating co-morbid precipitating causes. Successful treatment of DKA requires frequent monitoring of patients regarding the above goals by clinical and laboratory parameters. Table 2 below illustrates the initial management of patients with diabetic ketoacidosis^2,26^.

**Table 2:** Detailed management of patients with diabetic ketoacidosis

Fluid therapy	DKA is a volume-depleted state with an estimated 6 l of total body water deficit. Initial fluid therapy aims to increase intravascular volume and ensure sufficient urine flow. We advise isotonic saline should be administered as the first beverage at 15–20 ml/kg of body weight per hour or 1–1.5 l during the first hour. The choice of the fluid for additional replenishment depends on the degree of hydration, the level of serum electrolytes, and the output of the urine. Over 12–24 h, replacing half of the expected sodium and water deficit is appropriate. Hydrating fluid in the first hour of therapy before insulin administration provides time to obtain serum potassium. Osmotic diuresis and the modulation of counter-regulatory hormone secretion are the mechanisms for lowering blood sugar.
Insulin therapy	Insulin administered in physiologic doses is essential for DKA treatment. Administer an IV bolus of regular insulin (0.1 U/kg body weight) followed by a 0.1 U/kg/hr constant infusion of regular insulin. The insulin rate should be reduced to 0.05 U/kg/hr when plasma glucose hits 200–250 mg/dl. The insulin infusion rate should change to maintain blood glucose levels. Regular insulin is best administered via continuous IV infusion due to its short half-life and simple titration.
Potassium therapy	DKA patients with acidosis and insulinopenia have mild to moderate hyperkalemia due to acidosis and insulin depletion. Insulin therapy decreases serum potassium levels. Acidosis correction, volume enlargement, and potassium replacement are commenced when serum levels drop below 5.3 mmol/l.
Bicarbonate therapy	Bicarbonate therapy has neither a chance of benefit nor benefit in DKA, according to a prospective randomized study of patients with a pH between 6.9 and 7.1. Adult patients with pH 6.9 should be given 100 mmol sodium bicarbonate in 400 ml sterile water with 20 mmol KCl administered at 200 ml/h for 2 h until the pH rises to 7.0. Treatment continues every 2 h if necessary.
Phosphate therapy	Phosphate may be used in individuals experiencing adverse effects from hypophosphatemia, but may cause hypocalcemia when administered in large doses.
Nursing aspects of DKA	Nursing management is essential for patients with comatose or pre-comatose states, and regular maintenance of infusions, nasogastric tubes, CVP lines, urine catheters, ECG monitors, etc. Monitor temperature, pulse, blood pressure, respiration, and cognitive state every hour to confirm progress.

A patient with diabetic ketoacidosis might have normal potassium levels before the initiation of treatment; the medical practitioner should take caution to prevent severe hypokalemia after initiating insulin therapy. Table 3 shows endocrine society’s clinical practice guideline for hospitalized patients with diabetes^2,26–29^.

**Table 3:** Endocrine society’s clinical practice guideline for hospitalized patients with diabetes

In patients with impaired glycemic control should be on continuous glucose monitoring devices as they effectively prevent hypoglycemic crises.
Scheduled insulin therapy should be administered to in patients on glucocorticoid therapy or direct enteral nutrition should they develop hyperglycemia.
Outpatients on regular insulin pump therapy who are psychologically and physically fit may self-manage the device with the supervision of medical personnel.
Education of admitted diabetic patients on essential management of the condition aids in reasonable glycemic control after discharge and decreases the chance of hospital readmission.
Diabetic patients being scheduled for elective surgery are desired to have a preoperative HbA1c of <8% and an immediate preoperative blood glucose of <180 mg/dl as it improves the patient’s postoperative condition.
Carbohydrate-containing drinks should not be given to preoperative patients with diabetes.
Correctional insulin can be used as the sole initial management for hospital admissions of newly recognized hyperglycemia or well-controlled diabetes.
In patients previously on insulin therapy with a continuous blood glucose of >180 mg/dl should be placed on scheduled insulin therapy.
In some patients with type 2 diabetes with milder degrees of hyperglycemia, a Dipeptidyl peptidase inhibitor can be used with correction insulin, Provided there are no contraindications to using any of them.

This guideline addresses several crucial aspects of care precise for inpatient management of noncritically ill patients with diabetes or newly recognized hyperglycemia that can potentially improve clinical outcomes in the hospital and following discharge. This guideline addresses and updates some of the standards of care for glycemic management for noncritically ill-hospitalized adult patients with diabetes^30^.

## Conclusions

Diabetic ketoacidosis is often a severe medical emergency that results in a complication of diabetes. When the diagnosis of diabetic ketoacidosis is made, the blood glucose level is higher than 250 mg/dl, the bicarbonate level is less than 15 mmol/l, the arterial pH is less than 7.3, and there is ketonuria or ketonemia. It occurs primarily in patients with type 1 diabetes. The incidence is roughly two episodes per 100 patient-years of diabetes, with about 3% of patients with type 1 diabetes initially presenting with diabetic ketoacidosis. It can occur in patients with type 2 diabetes as well; however, this is less common. Diabetic ketoacidosis usually occurs due to absolute or relative insulin lack accompanied by increased glucagon, cortisol, growth hormone, and epinephrine. This insulin deficiency enhances hepatic gluconeogenesis, glycogenolysis, and lipolysis leading to severe hyperglycemia, ketoacidosis, and ketonuria.

In most cases, there are precipitating factors, which could be: underlying infection, missed insulin treatment, previously unknown diabetes, surgical stress, etcetera. Prompt diagnosis and treatment are essential to improve patient outcomes. Treatment involves fluid resuscitation, insulin administration to correct hyperglycemia, correction of acidosis, electrolyte imbalances, and treatment of underlying causes or precipitants. The prognosis of adequately treated patients is excellent.

## Ethics statement

It was exempted and waived at my institution.

## Patient consent

Patient consent was waived due to the minimal risk nature of the observational chart review study.

## Sources of funding

All authors have declared that they have no financial relationships at present or within the previous three years with any organizations that might have an interest in the submitted work.

## Authors’ contributions

The author and co-authors played important roles to actualize this article as shown below: C.E.: conceptualization, visualization, supervision, oversight and leadership, writing of the original draft; J.A.D.: conceptualization, validation, writing of the original draft, methodology; A.-O.U.: resources, software, visualization; E.O.E.: conceptualization, validation, resources, methodology; I.C.O.-C.: resource and journal review, formal analysis, writing of the original draft; L.O.U.: writing of the original draft, data creation, project administration; A.I.T.: writing of the article, data curation, leadership; J.O.I.: writing of the article, reviewing and revising the text; A.O. I.: project administration, editing, concept rephrasing; P.U.N.: literature search, writing, and editing; M.O.K.: editing, concept rephrasing, and editing; C.E.: drafting and revising the article; A.I.B.: concept rephrasing and maintenance of data research integrity; O.A.: preparation, typing, editing; O.S.O.: methodology and drafting of conclusion; F.O.J.: project administration, review, and editing; H.I.R.-Y.: editing, concept rephrasing, and editing.

## Conflicts of interest

In compliance with the ICMJE uniform disclosure form, all authors declare the following: Payment/services info: All authors have declared that no financial support was received from any organization for the submitted work.

## Research registration unique identifying number (UIN)

None.

## Guarantor

Chukwuka Elendu, E-mail: elenduchukwuka@yahoo.com.

## Provenance and peer review

Commissioned, externally peer reviewed.

## Data availability statement

Data will be made available by the authors upon reasonable request.

## Institutional review board statement

The study was conducted in accordance with the Declaration of Helsinki and approved by the Ethics Board of the Mayo Clinic (IRB ID: 21-007698).

## Informed consent statement

Patient consent was waived due to the minimal risk nature of the observational chart review study.
